# Social and Psychological Influences on Emerging Adult Drinking Behavior

**Published:** 2004

**Authors:** Helene Raskin White, Kristina Jackson

**Affiliations:** Helene Raskin White, Ph.D., is a professor at the Center of Alcohol Studies, Rutgers, The State University of New Jersey, in Piscataway, New Jersey. Kristina Jackson, Ph.D., is a research assistant professor in the Department of Psychological Sciences, University of Missouri–Columbia, Columbia, Missouri

**Keywords:** young adult, young adulthood, undergraduate student, drinking behavior, AOD (alcohol and other drug) use, abuse and dependence, heavy drinking, AOD use pattern, causes of AODU (alcohol and other drug use), AOD effects and consequences, AODR (alcohol and other drug related) interpersonal and societal problems, intervention, prevention, social costs and benefits of AOD, social behavior, perception of norms

## Abstract

Emerging adulthood, the transitional period between high school and young adulthood, is marked by the formation of identity, the establishment of more mature interpersonal and intimate relationships, and the transition to new adult-type roles. It also is a time of increased alcohol use and abuse, which can have long-term effects on both physical and psychological well-being and may have implications for the attainment of traditional adult roles. Gender, race/ethnicity, marital status, college, employment, peer and family influences, individual temperament, and attitudes about drinking all influence drinking behavior in this population. Attending college may represent a special risk to emerging adults, as increases in alcohol availability and acceptance of drinking on college campuses may lead to increases in heavy drinking among students. The nonstudent population of emerging adults also is an important target for preventive interventions, especially because people in this segment of the population may be less likely to mature out of heavy drinking patterns established during adolescence. Thus, the transition from high school to young adulthood appears to be an ideal developmental turning point during which to target interventions.

“Arnett (2000) referred to the transitional period from high school to young adulthood as emerging adulthood.” This stage of life is defined as the period from the end of secondary school through the attainment of adult status (Arnett 2005), covering approximately ages 18 to 25, although it can extend longer. Emerging adulthood is marked by frequent change and exploration. It also is a period of increased alcohol use and abuse (Arnett 2000; Bachman et al. 1997).

This article examines the developmental changes and related drinking patterns and problems that occur during emerging adulthood and the sociodemographic and psychosocial factors that influence drinking in this population. It concludes with a discussion of the implications of this research for prevention.

## Emerging Adulthood

Numerous historical changes have caused emerging adulthood to become a distinct period in the life course in industrialized countries. One major contributor is the fact that people are waiting longer before marrying. In the United States, the median age of marriage increased from 20 for women and 22 for men in 1950 to 25 for women and 27 for men in 2000 (Arnett 2005). Several reasons may account for this delay in marriage, including more people seeking college and postgraduate education, the invention of the birth control pill and changing standards of sexual morality, changes in women’s roles, and an increased desire for independence and freedom among youth (Arnett 2005).

Emerging adulthood is marked by a variety of developmental tasks, including identity formation and the establishment of more mature interpersonal and intimate relationships (Arnett 2000; Schulenberg and Maggs 2002). During this period, young people also obtain the education and training needed for future careers. These tasks must be completed to make a successful transition to adulthood, and failure to master them can result in frustration and stress, which can lead to a variety of unhealthy behaviors, including increased alcohol use. Paradoxically, alcohol use can impede the successful mastery of these developmental tasks and may exacerbate failures and increase stress (Schulenberg et al. 2003).

Emerging adulthood often is characterized by changes in residence, employment or education, and romantic relationships. It is a time of identity exploration and self-focus; initiation of new roles; development of new social networks; separation from families and old friends; increased choices and opportunities; increased independence; freedom from time constraints and social control; and decreased parental support, guidance, and monitoring (Arnett 2005; Schulenberg and Maggs 2002).

Arnett (2005) has suggested that increases in drinking during emerging adulthood are normative in Western society because of the many developmental changes taking place at this age. Social control lessens during this period, and people become freer to choose behaviors (e.g., heavy drinking) and lifestyles that are not constrained by others. Along with identity exploration and trying out various behaviors, emerging adults may seek out the altered states of consciousness that different substances can induce. In addition, constructing a stable identity can be confusing and difficult, and some emerging adults may use substances to relieve their identity confusion. Emerging adulthood also is a period of instability, and people often are involved in unstable social networks. Clearly, no one set of norms governs drinking behavior. People are free to make their own decisions independently and to do things that may not be acceptable in either adolescence or young adulthood,^1^ including using illegal substances. In sum, the developmental changes taking place during emerging adulthood can lead to increased drinking as a means of dealing with greater stress or as a result of the increased freedom that allows emerging adults to drink heavily. In addition, the weakening of parental monitoring and increased importance of peer relationships can lead to increased alcohol consumption (Borsari and Carey 2001) (see the section “Peer Influences,” below). Finally, in the United States, at age 21 people can legally obtain and consume alcohol, which contributes to increased access and opportunities to use alcohol.

## Drinking Patterns and Problems During Emerging Adulthood

Many people begin to use alcohol before they graduate from high school. However, peak use of alcohol occurs during emerging adulthood, and this excessive drinking appears to be normative behavior. In North America and many other industrialized societies, binge^2^ or excessive drinking during emerging adulthood is condoned, and perhaps even encouraged, particularly for those attending college (Arnett 2005). Data from the Monitoring the Future (MTF) study across several years indicate that 30-day prevalence (use in the last 30 days) peaks at ages 21–22 (at 85 percent for men and 76 percent for women), and heavy drinking (drinking five or more drinks in a row in the past 2 weeks) peaks at ages 21–22 for men (at 55 percent) and at ages 19–20 for women (at 33 percent) and then declines linearly into young adulthood (Bachman et al. 1997).

**Alcohol Use Among Emerging Adults**

- Among people ages 21–22, 85 percent of men and 76 percent of women have used alcohol within the last 30 days (Bachman et al. 1997).
- 55 percent of men ages 21–22 and 33 percent of women ages 19–20 drank five or more drinks in a row in the past 2 weeks (Bachman et al. 1997).
- People ages 18–29 have the highest rates of past-year alcohol abuse and dependence (Grant et al. 2004).

Drinking during emerging adulthood can serve positive functions, such as facilitating friendship formation, but people also experience high rates of alcohol-related problems during this period. For example, people ages 18 to 29 have the highest rates of past-year alcohol abuse and dependence (Grant et al. 2004). Alcohol-related problems during emerging adulthood can have long-term effects on physical and psychological well-being (Schulenberg et al. 2003) and can have implications for the attainment of traditional adult roles (e.g., heavy drinking leads to poor academic outcomes, which in turn lead to less favorable occupational opportunities). Numerous studies have identified problems related to alcohol use, including fatal and nonfatal injuries and overdoses, academic/vocational failures, violence and other crime, unintended pregnancies, and sexually transmitted diseases (Perkins 2002*a*; Hingson et al. 2002), although the extent to which alcohol use causes some of these problems (e.g., academic problems) remains unclear (Jackson et al. 2005). Rather, alcohol use and problems during emerging adulthood may be the result of some common factor (such as lower academic motivation, in the case of academic problems) (Schulenberg et al. 2003). Impaired driving, another serious problem associated with drinking, is especially prevalent among emerging adults (Hingson et al. 2002). In addition, about half of college students report past-year hangovers, nausea, and vomiting as a result of drinking, and about one-fourth report blackouts (or memory loss while intoxicated) (Jackson et al. 2005). Furthermore, excessive drinkers can create problems for others, including physical and sexual assaults, insults and humiliation, preventing others from studying/sleeping, and vandalism (Jackson et al. 2005; Perkins 2002*a*).

## Changes in Drinking During Emerging Adulthood

The transition out of high school may be marked by increases in alcohol use and intoxication (Baer et al. 1995; White et al. 2005). Even men who drank heavily in high school may drink more and become intoxicated more often after high school (Baer et al. 1995). Drinking patterns during the senior year of high school generally are useful in predicting post–high school drinking behavior, although research results vary (Bachman et al. 1997). Some studies have found a high degree of individual stability in problem drinking from the early twenties into adulthood (Jackson et al. 2001), whereas others have not (e.g., Temple and Fillmore 1986).

Most emerging adults will outgrow heavy drinking and related problems before adulthood, on their own and without treatment (Marlatt et al. 1998). (For more detail on changes in drinking during this period, see this issue’s article by Maggs and Schulenberg, and for a discussion of maturing out of alcohol use, see the sidebar by O’Malley.)

**Sociodemographic Characteristics Related to Drinking Among Emerging Adults**

***Gender***

- Among 19- to 30-year-olds:
  – 45 percent of men and 26.7 percent of women reported heavy drinking in the past 2 weeks (Johnston et al. 2004).
  – 7 percent of men and 3 percent of women reported daily drinking (Johnston et al. 2004).

***Race/Ethnicity***

- Among emerging adults, Whites and Native Americans drink the most, African Americans and Asians drink the least, and Hispanics are in the middle range (Caetano and Kaskutas 1995).
- Drinking peaks among Whites at ages 19–22; heavy drinking peaks later and lasts longer into adulthood among African Americans and Hispanics (Caetano and Kaskutas 1995).

***Marital Status and Parenthood***

- Being married had a strong effect on drinking behavior after high school graduation.
  – Married women had the greatest decreases in drinking behavior. Married men had fewer increases in drinking than people in all other types of living arrangements (Bachman et al. 1997).
- Being engaged had an influence on drinking behavior similar to that of being married (Bachman et al. 1997).
- Being a parent is related to reduced alcohol use for both men and women, although a large part of this effect is a result of being married (Bachman et al. 1997).
- Most women who become pregnant eliminated their alcohol use, although most of their husbands did not (Bachman et al. 1997).
- Getting divorced led to increased drinking (Bachman et al. 1997).

***College Status***

- Heavy drinking and related problems are pervasive in the early twenties regardless of college attendance (Jackson et al. 2005; White et al. 2005).
- College students and nonstudents had similar quantity and frequency of drinking, number of times intoxicated, and rates of drinking problems, but college students matured out of drinking more quickly than nonstudents (White et al. 2005).
- College students had lower rates of daily drinking than their noncollege peers, but when they did drink, they tended to drink greater amounts than nonstudents (O’Malley and Johnston 2002).
- College students had higher rates of current drinking (within the last 30 days) (O’Malley and Johnston 2002).
- Rates of alcohol dependence were lower for college students than for 18- to 24-year-olds in the general population (Jackson et al. 2005).
- College students reported lower rates of heavy drinking while in high school than nonstudents, but increased their use in college to higher levels than nonstudents (Bachman et al. 1997).
- Differences in drinking between college students and nonstudents were fully accounted for by background characteristics and living arrangements (Bachman et al. 1997).

***Employment***

- Full-time civilian employment after high school was related to slight increases in drinking within the past 30 days and to slight decreases in heavy drinking (Bachman et al. 1997).
- Joining the military after high school was associated with greater than average increases in current drinking and heavy drinking (Bachman et al. 1997).
- Unemployed men, but not women, significantly reduced their drinking (Bachman et al. 1997).
- Homemakers reduced their current and heavy drinking, but this change can be accounted for by their marital and parental status rather than by the homemaker role (Bachman et al. 1997).

## Sociodemographic Correlates of Heavy Drinking Among Emerging Adults

Sociodemographic characteristics—including gender, race/ethnicity, marital status, parenthood, college status, and employment—all influence drinking patterns among emerging adults, as discussed in the following sections.

### Gender

Research consistently shows that most indexes of alcohol use, and especially heavy drinking, are higher among males than females (Johnston et al. 2004; O’Malley and Johnston 2002). Of the 19- to 30-year-olds in the MTF study, 45 percent of men and 26.7 percent of women reported heavy drinking (defined in that study as five or more drinks on one occasion) in the past 2 weeks, and 7.4 percent of men and 3 percent of women reported daily drinking. In addition, the gender disparity in heavy drinking increases between late adolescence (i.e., senior year of high school) and young adulthood (Johnston et al. 2004). In contrast, the rates of alcohol problems among male and female college students tend to converge (Jackson et al. 2005), although men still report more problems in the public domain (e.g., public risk-taking, aggression, legal problems, and problems that endanger others) compared with women.

### Race/Ethnicity

Racial and ethnic differences in drinking and related problems have been documented in the literature. In general, White and Native American emerging adults drink more than African Americans and Asians, and drinking rates for Hispanics fall in the middle. In addition, in contrast to the peak in drinking among Whites around ages 19–22, heavy drinking among African Americans and Hispanics peaks later and persists longer into adulthood (Caetano and Kaskutas 1995). Caetano and Kaskutas suggest that this ethnic difference results, in part, from the fact that Whites see heavy drinking as part of a youthful lifestyle, whereas Hispanics tend to see heavy drinking as a “right” they earn when they reach maturity.

### Marital Status and Parenthood

Bachman and colleagues (1997) examined changes in heavy drinking (defined in that study as five or more drinks in one sitting in the last 2 weeks) and in current drinking (defined as use in the past 30 days) from the senior year of high school into the early twenties. These researchers found that being married had a strong effect on changes in drinking behavior after high school graduation even when they controlled for other relevant factors. Married women had the greatest decreases in drinking behavior, and married men, compared with men in all other categories of living arrangements (i.e., cohabiting or living with parents, in a dormitory, alone, or in other arrangements), showed the fewest increases. The data also indicated that becoming engaged (i.e., making a commitment to a relationship) had a similar but less powerful effect on drinking compared with marriage, whereas becoming divorced led to increased drinking behavior. Drinking rates among cohabitants did not decline as much as they did in married people; however, drinking also did not increase as much as it did among those who were unmarried. It should be noted, however, that the cohabitants were the heaviest drinkers in high school.

Being a parent also was related to reductions in alcohol use for both men and women, although a large part of this effect was simply a result of getting married. Most women who became pregnant eliminated their alcohol use, although most of their husbands did not (Bachman et al. 1997).

### College Status

Some argue that the college campus environment itself encourages heavy drinking (Toomey and Wagenaar 2002). Alcohol use is present at most college social functions, and many students view college as a place to drink excessively. Students experience greater exposure to drinking and encounter higher levels of peer drinking and positive attitudes toward alcohol as they transition from high school to college (Borsari and Carey 2001).

Although much of the research targeting emerging adulthood has focused on college students, several studies have noted that heavy drinking and related problems are pervasive among people in their early twenties, regardless of college attendance (Jackson et al. 2005; White et al. 2005). Comparing several national data sets, O’Malley and Johnston (2002) reported that college students drink less frequently than their noncollege peers (i.e., they report lower rates of daily drinking). However, when students do drink, they tend to drink in greater quantities than nonstudents. In addition, college students report higher rates of current drinking (past 30 days).

**Figure 1:**
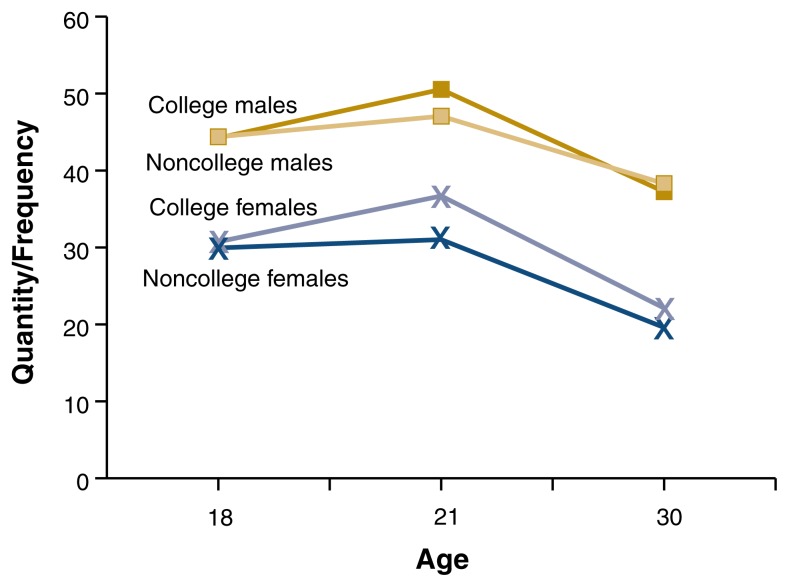
Changes in alcohol use over time for college and noncollege men and women. Quantity and frequency of drinking in the past year did not differ significantly for college students and their nonstudent peers at any age during emerging adulthood. SOURCE: White et al. 2005.

**Figure 2:**
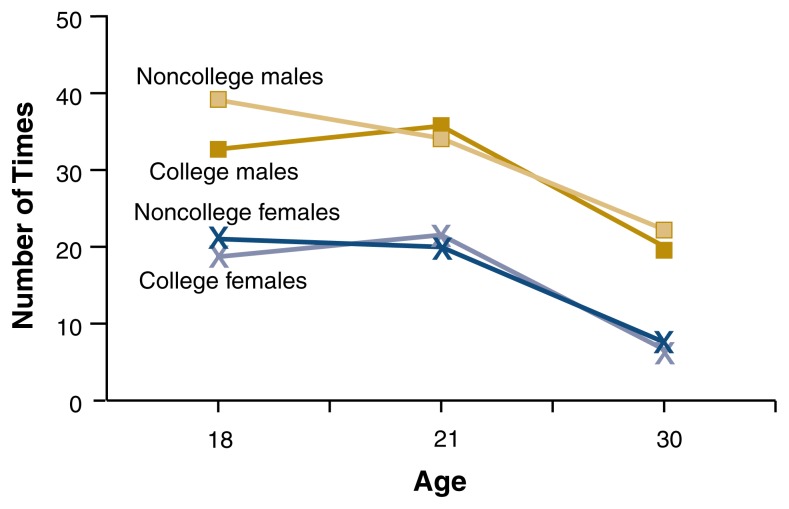
Changes in the number of times college and noncollege men and women were intoxicated. The number of times intoxicated in the past year did not differ significantly for college students and their nonstudent peers at any age during emerging adulthood. SOURCE: White et al. 2005.

**Figure 3:**
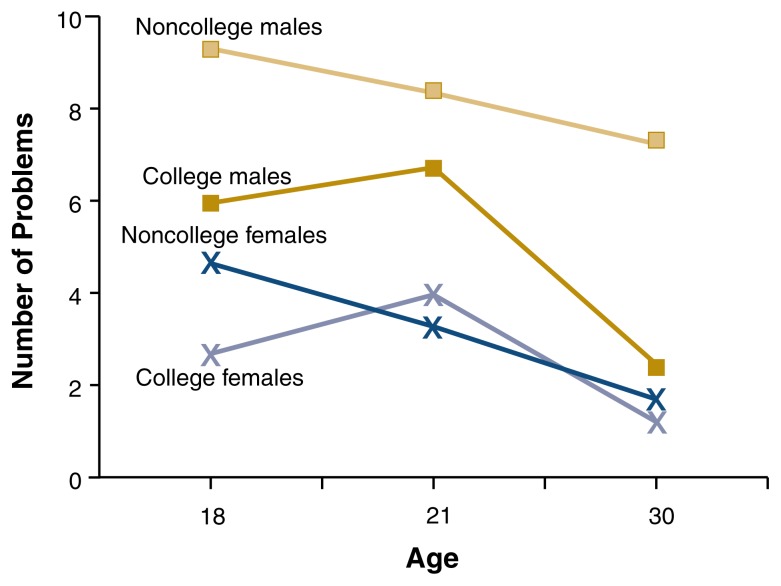
Changes in alcohol-related problems over time for college and noncollege men and women (alcohol users only). Rates of drinking problems (negative consequences related to alcohol use) did not differ significantly for college students and their nonstudent peers at age 21 (i.e., during emerging adulthood). However, noncollege peer drinkers reported significantly higher levels of alcohol-related problems in late adolescence (age 18) and young adulthood (age 30) than college student drinkers. SOURCE: White et al. 2005.

In contrast, White and colleagues (2005) found that quantity and frequency of drinking in the past year, number of times intoxicated in the past year, and rates of drinking problems (the frequency of experiencing negative consequences related to alcohol use) in the past year did not differ significantly for college students and their nonstudent peers during their early twenties (i.e., emerging adulthood) (see figures 1–3). However, noncollege drinkers reported higher levels of alcohol-related problems in late adolescence (age 18) and young adulthood (age 30) than college student drinkers. In other words, students matured out of drinking problems more quickly than nonstudents. Rates of alcohol dependence diagnosis appear lower for college students than for 18- to 24-year-olds in the general population (Jackson et al. 2005). Further, people in their thirties who did not go to college reported a higher prevalence of heavy drinking episodes (defined as drinking six or more drinks on one occasion during the last 30 days) than people who did go to college (Muthén and Muthén 2000).

Bachman and colleagues (1997) found that college students, compared with their nonstudent peers, reported lower rates of heavy drinking while in high school but then increased their use in college to higher levels than their nonstudent peers. The authors concluded that living arrangements particularly contributed to the college effect. For example, living in dormitories increased the risks for heavy drinking, whereas living with a spouse (i.e., being married) decreased the risks. Overall, the drinking differences between college students and nonstudents were fully accounted for by background characteristics and by where and with whom people lived.

### Employment

Bachman and colleagues (1997) reported that people who obtained full-time civilian employment after high school showed a slight increase in current drinking (past 30 days) and a slight decrease in heavy drinking. In contrast, those who joined the military reported greater than average increases in current drinking and in heavy drinking. These changes persisted when other variables were controlled, suggesting that the military experience contributes to increased alcohol use. Unemployed men, but not women, significantly reduced their drinking. Homemakers also reduced their current and heavy drinking, but the authors suggest that this change was a result of their marital and parental status rather than the role of being a homemaker.

## Psychosocial Correlates of Heavy Drinking Among Emerging Adults

Many psychological and social factors influence alcohol use among emerging adults. As described in this section, these factors have been studied most often in college students.

### Impulsivity, Sensation-Seeking, and Risk-Taking

One of the most consistent predictors of substance use among adolescents and emerging adults is sensation-seeking, defined as the pursuit of novel and intense experiences (Arnett 2005). In fact, sensation-seeking increases as young people develop from adolescents into emerging adults. Measures of impulsivity and sensation-seeking among emerging adults have been related to higher frequency and quantity of drinking and to experiencing more negative alcohol-related consequences (Baer 2002; Jackson et al. 2005). Sensation-seeking and impulsivity also have been linked to deviant behavior and nonconformity, both of which are predictors of heavy drinking and related problems among youth (Baer 2002).

Optimism is an almost universal trait among emerging adults (Arnett 2005). Because of their optimistic bias, many emerging adults do not see themselves as vulnerable to any negative consequences that might occur because of drinking, such as having an accident or becoming dependent on alcohol. Thus, emerging adults are more likely to take risks and to drink excessively, although risk-taking may not be the impetus for their drinking. In other words, the decision to drink is more influenced by the perceived benefits of drinking (i.e., positive expectations of the effects of alcohol) than by the perceived risks (Goldberg et al. 2002).

### Negative Affect

The findings regarding the association between negative mood and problematic alcohol use during emerging adulthood have been inconsistent (Jackson et al. 2005). Most studies examining this association have relied on samples of college students, who report relatively better levels of overall mental health than do nonstudents (Baer 2002). Nevertheless, extremely high levels of negative affect, as seen in anxiety disorders, are associated with problem drinking in college students (Jackson et al. 2005). Jackson and Sher (2003) found that alcohol use disorders were associated with psychological distress, as measured by past-week physiological and psychological functioning and symptoms, among 18- to 29-year-olds who functioned well in most areas of their lives.

Research has suggested that some people drink to regulate emotional distress. In support of this, Cooper and colleagues (2000) found that drinking to cope with negative affect predicted heavy drinking (a composite of drinking to intoxication and drinking five or more drinks) as well as drinking problems in 19- to 25-year-olds. However, emerging adults are more likely to drink for “positive” or celebratory reasons than to drink to cope with negative feelings (Read et al. 2003).

### Alcohol Expectancies

Positive expectancies about alcohol’s effects play a key role in the drinking behavior of emerging adults. With age, adolescents increasingly expect benefits from drinking and become less convinced of the risks (Schulenberg and Maggs 2002; Smith et al. 1995). However, one study that followed college students over 3 years found a decline in positive expectancies, whereas alcohol use rates remained stable (Sher et al. 1996).

Expectancies appear to predict both drinking initiation in adolescence and maintenance of drinking throughout young adulthood. There has been less evidence that expectancies predict drinking problems across adolescence and young adulthood (Jones et al. 2001). As with older adults, drinking for social purposes is associated with greater consumption, and drinking for escape or relief is associated with problem drinking (Jackson et al. 2005). In addition, among college students, heavier drinkers endorse an overall greater number and variety of reasons for drinking (Carey and Correia 1997).

### Peer Influences

People entering college or the workforce may be especially vulnerable to the influence of peers because of their need to make new friendships. They may increase their drinking to facilitate peer interactions. Borsari and Carey (2001) maintain that peer influence is exerted directly (e.g., overt drink offers or urges to drink) and indirectly (e.g., modeling perceived social norms).

Although all these influences affect drinking behavior, one of the strongest correlates of drinking among emerging adults, and the subject of the most research on this topic, is perceived norms (Jackson et al. 2005). Many college students may drink more because of their misperceptions about the norms of drinking on their campuses. They may think campus attitudes are much more permissive toward drinking than they are and believe other students drink much more than they actually do (Borsari and Carey 2001, 2003; Perkins 2002*b*). Borsari and Carey (2003) compared descriptive norms (i.e., perception of others’ drinking behavior) and injunctive norms (i.e., perceived approval of drinking) and found students were more likely to overestimate approval of drinking among their peers than to overestimate their peers’ actual drinking behavior. Recent research has shown that addressing misperceptions of these norms, especially descriptive norms (Borsari and Carey 2001; Neighbors et al. 2004), has some success in reducing drinking (Perkins 2002*b*). It is worth noting, however, that a young person is just as likely to select a peer group based on the group members’ drinking behavior as he or she is to be influenced by peers to change his or her own drinking (Bullers et al. 2001).

### Family Influences

During emerging adulthood, parental monitoring decreases, and parents therefore have less influence on drinking patterns than do peers. Nevertheless, young people’s relationships with their parents continue to play a major protective role in development during this stage of the life cycle (Schulenberg and Maggs 2002). Parental drinking patterns have been shown to affect drinking by offspring over the life course (White et al. 2000). Young people model their own behavior on their parents’ patterns of consumption (including quantity and frequency), situations and contexts of use, attitudes regarding use, and use expectancies. The structure and environment of the family unit, as well as parent–child relationship attributes (e.g., parenting style, attachment and bonding, nurturance, abuse or neglect, conflict, discipline, and monitoring), have been found to correlate with adolescent alcohol use (White et al. 2000).

In addition, alcohol problems tend to aggregate in families (White et al. 2000). This family transmission can reflect genetics and/or modeling. Findings have been inconsistent about whether drinking patterns and problems during emerging adulthood differ between children of alcoholics and others (Jackson et al. 2005). There are several possible explanations for these equivocal findings. First, studies vary greatly in their measures of family history of alcoholism. Second, this research is based primarily on college students, and children of alcoholics who are at greater risk for alcohol problems may not enroll in college (Baer 2002). Finally, the peak in heavy drinking, which tends to occur when people reach their early twenties, may obscure differences between children of alcoholics and others. However, research using stringent criteria indicates that people with a family history of alcoholism are less likely than those with no family history to mature out of heavy drinking as they approach young adulthood (Jackson et al. 2001). In addition to parents, siblings can influence drinking through modeling, direct social influence, and access (Schulenberg and Maggs 2002).

## Summary

In sum, during the transition from adolescence to young adulthood, many people initially increase, and then decrease, their alcohol use. Bachman and colleagues (1997) suggest that increased drinking during the early part of emerging adulthood (from ages 18 to 22) results from changes in living arrangements (i.e., leaving parents and moving into dormitories or other housing shared with other emerging adults) as well as the freedom to purchase alcohol. Drinking during this time is culturally normative for most, although a minority of emerging adults will have drinking patterns that do not run true to this course—either they abstain completely or their drinking continues to increase after they reach adulthood. The decline in heavy drinking that occurs from the middle of emerging adulthood into young adulthood primarily results from increased responsibilities associated with marriage, parenthood, and career.

For the most part, the key factors that influence drinking in emerging adulthood are similar to those which influence adolescent drinking, although peer influence becomes increasingly more important than parental influence as the child ages, as do freedom from social control and stress related to attaining adult roles. Thus, moving out of the constraints of high school and away from parents makes emerging adulthood a stage of the life cycle in which people are at high risk for heavy drinking and alcohol-related problems. Maladaptive drinking behavior, in turn, may delay the accomplishment of developmental milestones, creating a self-defeating cycle for some. More research is needed about the intermediate and long-term effects of alcohol use during emerging adulthood, as well as the intrapersonal factors that facilitate or impede maturation out of heavy drinking.

## Implications for Prevention Programs and Interventions

Because some emerging adults will maintain or increase their problematic alcohol use over time rather than mature out of heavy drinking and related problems (Jackson et al. 2001), it is important to intervene with emerging adults before they develop long-lasting alcohol use patterns or disorders. Given that more than half of their college sample reported drinking to get drunk or “high” during their freshman year, Jackson and colleagues (2001) suggested that it is important to implement interventions prior to or upon college entry. Such interventions should focus on reducing the harms associated with heavy drinking (e.g., Marlatt et al. 1998). Universal screening is recommended to identify high-risk students and refer them to appropriate interventions. Recent advances in Internet-based screening have made it possible to screen large numbers of students for potential risk and provide them with immediate feedback on their drinking and risks, peer norms, and techniques to reduce risk. These interventions have been found to change perceived norms and reduce alcohol consumption (e.g., Neighbors et al. 2004), although more research is needed.

Several studies indicate that the nonstudent population of emerging adults is an important target for preventive interventions, especially because people in this segment of the population may be less likely to mature out of heavy drinking patterns established during adolescence (Muthén and Muthén 2000; White et al. 2005). Nonstudents’ risks for alcohol-related problems in their early twenties are as high as students’ risks. Unlike students, however, the risks for nonstudents appear to increase over time. This population does not have the benefits of campus health care centers or institutionally based programs that college students have. Similarly, students who do not live on campus may not have the benefits of campus substance use prevention programs (which often are designed for residents) or the protective benefits of campus organizations and peer groups. Intervening with emerging adults as they make the transition out of high school will ensure that interventions reach people who would not otherwise receive them. The period following high school graduation is an ideal time for interventions intended to prevent the problems that may result from such escalations in use, given that alcohol use increases considerably around this time (Jackson et al. 2001). Because most of the research on drinking among emerging adults has focused on college students, more research on nonstudent populations is needed to better inform the design of appropriate prevention efforts.

## Footnotes

1 Young adulthood is the period following emerging adulthood. It begins with the adoption of adult roles, such as marriage, parenthood, and a stable occupational path. It generally begins in the late twenties and continues through the thirties (Arnett 2000).

2 Binge drinking is defined by the National Institute on Alcohol Abuse and Alcoholism as consuming five or more drinks for men, or four or more drinks for women in about 2 hours.
